# Supplementary material for: Online repositories of photographs and videos provide insights into the evolution of skilled hindlimb movements in birds
Source: Commun Biol. 2023 Aug 15;6:781. doi: 10.1038/s42003-023-05151-z (PMC10427617; doi:10.1038/s42003-023-05151-z)
Supplement: Supplementary file 2 — Supplementary Materials [file 42003_2023_5151_MOESM2_ESM.pdf]

Supplementary Materials

Online repositories of photographs and videos provide insights into the evolution of skilled hindlimb movements in birds

Cristián Gutiérrez-Ibáñez*[1], Clara Amaral-Peçanha*[2], Andrew N. Iwaniuk[4], Douglas R. Wylie[1], Jerome Baron[2,3].

[1] Department of Biological Sciences, University of Alberta, Edmonton, Canada.

[2] Graduate Program in Physiology and Pharmacology and [3] Department of Physiology and Biophysics, Institute of Biological Sciences, Federal University of Minas Gerais, Belo Horizonte, Brazil

[4] Department of Neuroscience, Canadian Centre for Behavioural Neuroscience, University of Lethbridge, Lethbridge, Alta., Canada

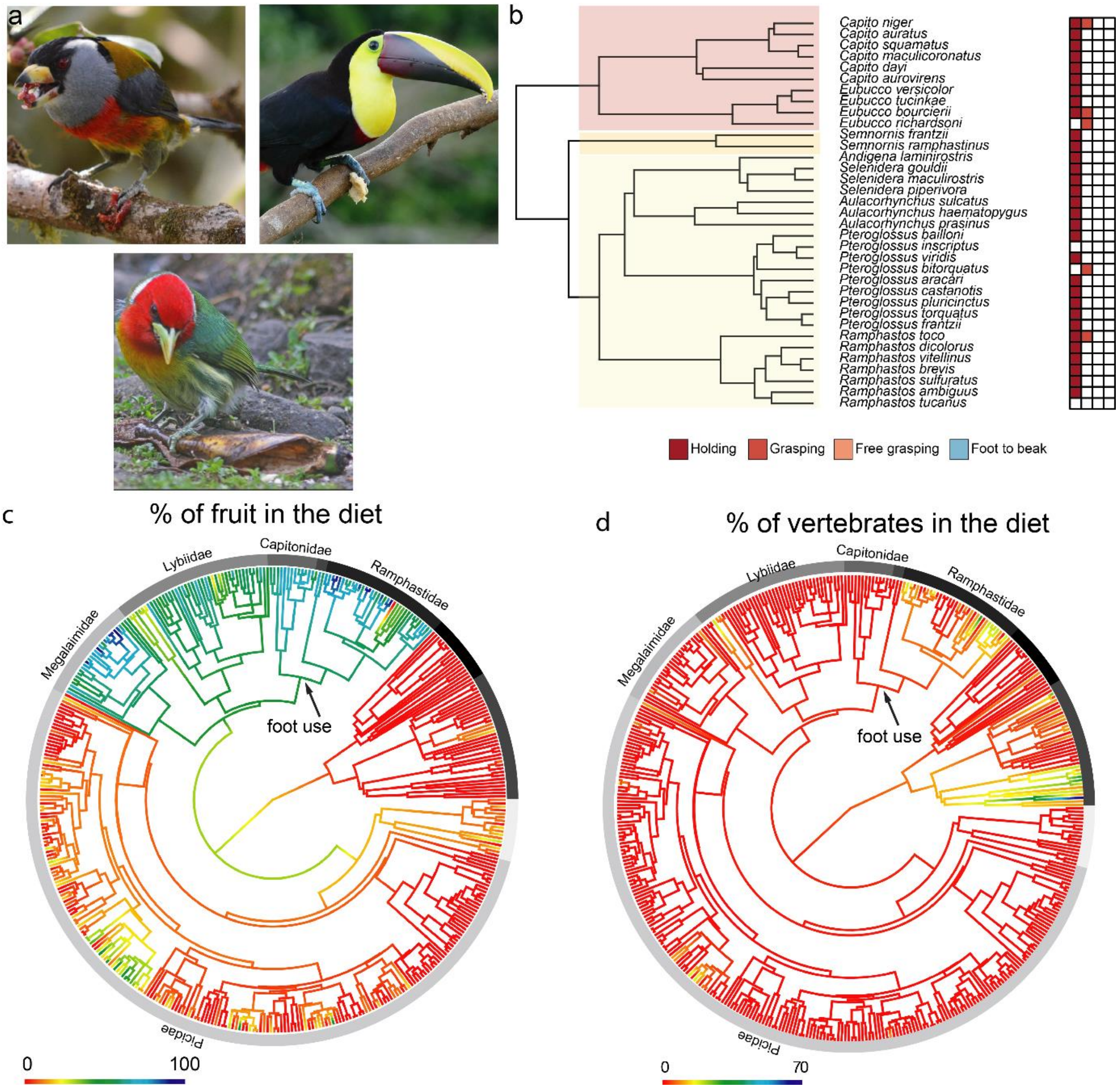

Supplementary figure 1. Foot use evolution in new world barbets and toucans. a, examples of foot use in the three families of Piciformes where foot use is present, Semnornithidae (Toucan-Barbets. Top left: Toucan Barbet, *Semnornis ramphastinus*), Rhamphastidae (Toucans and toucanets, top righ: Yellow-throated Toucan, *Ramphastos ambiguous*), and Capitonidae (New world barbets, bottom panel: Red-headed Barbet, *Eubucco bourcierii*). Photographer credits are listed in Table S7. **b,** a character matrix species level phylogenies for the same three families. Grasping is rare (only recorded in five species) and most species hold objects against a perch. **c** and **d** show ancestral state reconstruction for all species of Piciformes with the percentage of the diet that is constituted by fruits (c) and vertebrates (d) mapped. The node where foot use is likely to have evolved is indicated.

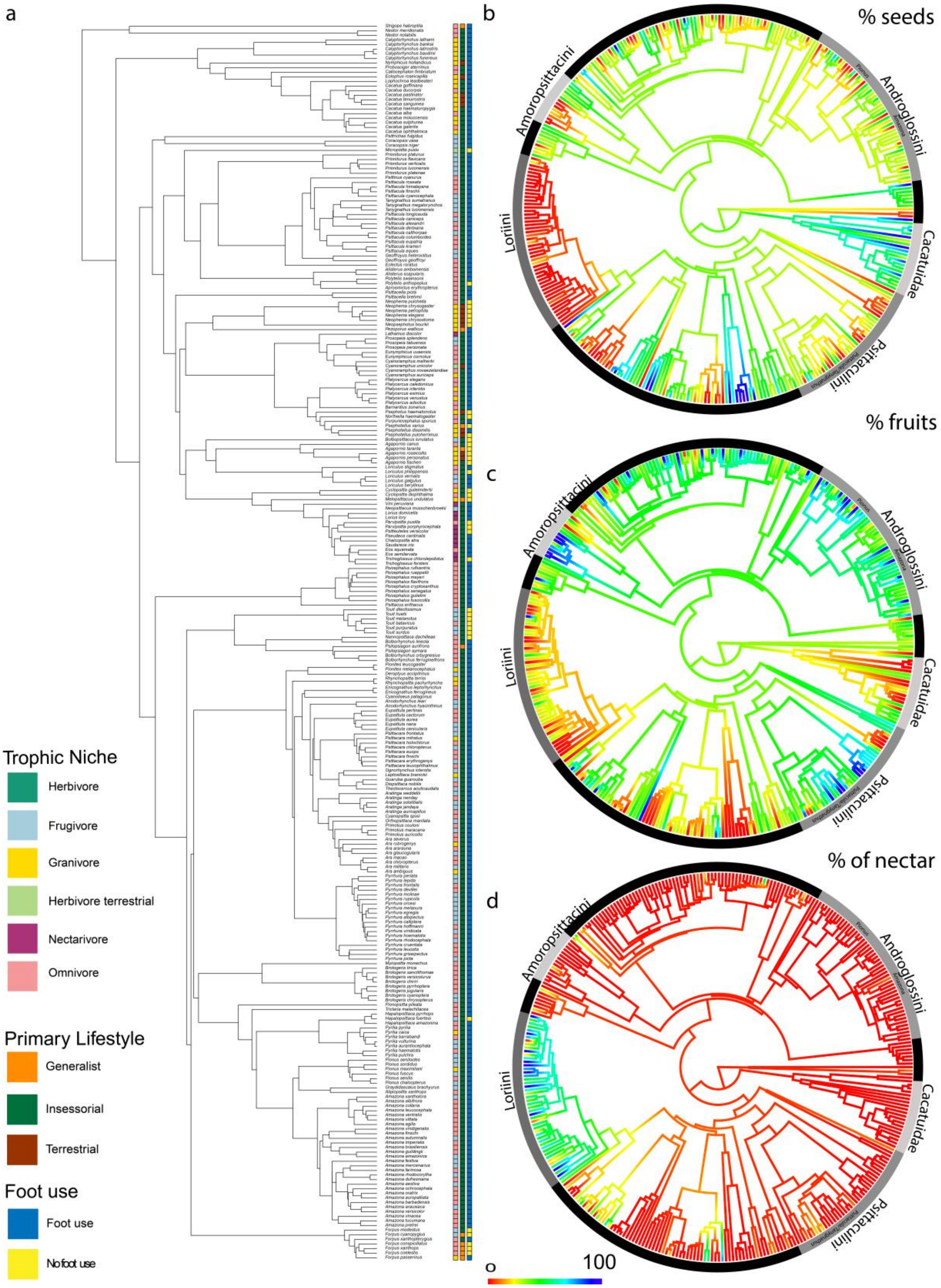
a
b
% seeds
c
% fruits
d
% of nectar
Amoropsittacini
Androglossini
Loriini
Cacatuidae
Psittaculini
Trophic Niche
Herbivore
Frugivore
Granivore
Herbivore terrestrial
Nectarivore
Omnivore
Primary Lifestyle
Generalist
Insessorial
Terrestrial
Foot use
Foot use
No foot use
0
100

Supplementary figure 2. Interplay between ecological and trophic characteristics and foot use among parrots. A, character matrix showing habitat, trophic niche, primary lifestyle and foot use in 282 species of parrots. **b**, **c**, and **d**, show ancestral state reconstruction for all species of parrots of the percentage of the diet that is constituted by seeds (b), fruits (c) and nectar. (d).

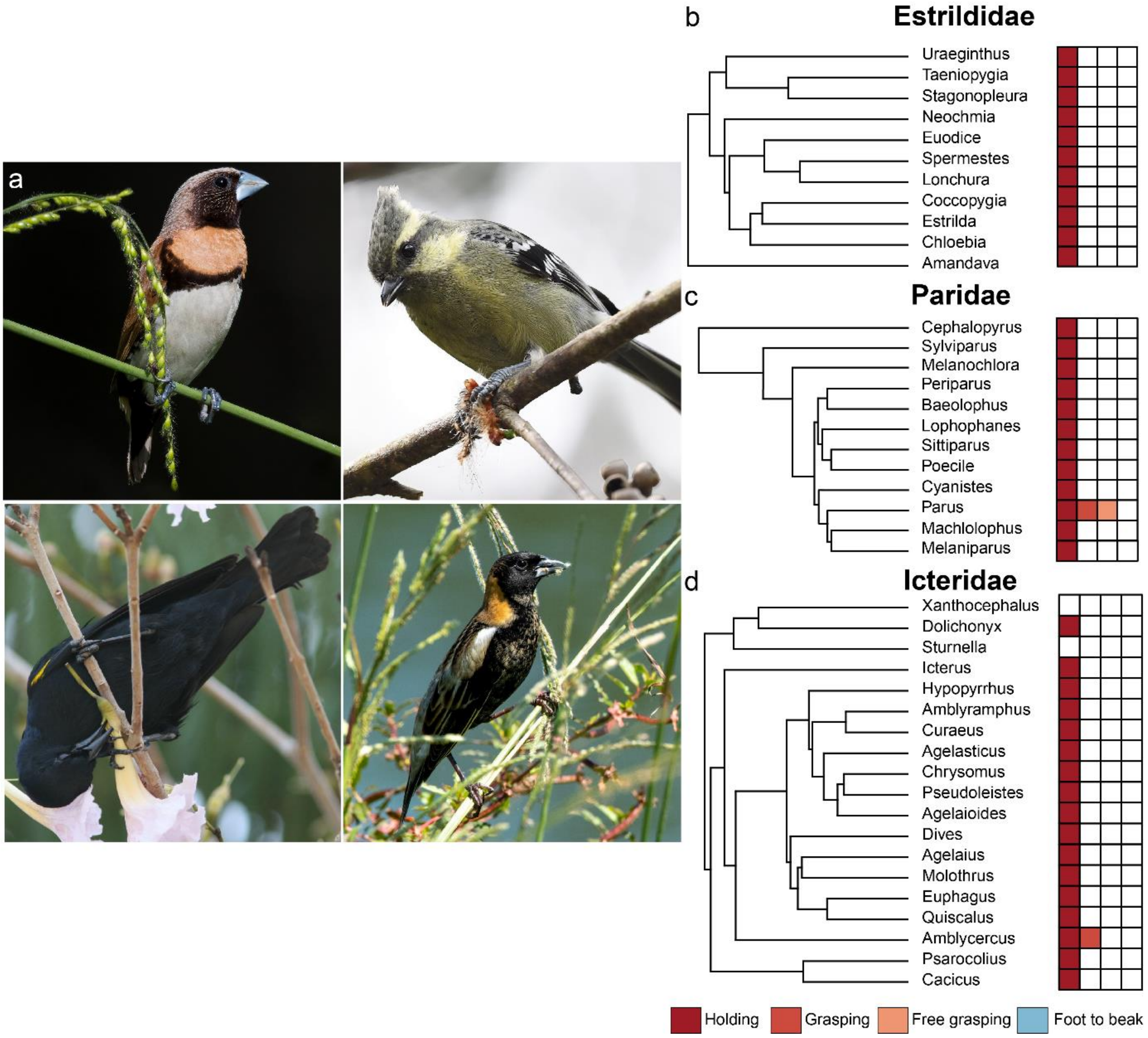

Supplementary figure 3: foot use three families of Passeriformes. **a**, shows examples of members of the families estrildid (top left: Chestnut-breasted Munia, *Lonchura castaneothorax*), Paridae (top right: Indian Yellow Tit, *Machlolophus aplonotus*) and Icteridae (bottom two panels, left: Yellow-shouldered Blackbird, *Agelaius xanthomus*; right: Bobolink, *Dolichonyx oryzivorus*) using their feet to manipulate objects. Photographer credits are listed in Table S7. **b, c** and **d** show a character matrix at the genus level for the same families. colored squares reflect the presence of each of the four behavioral elements.

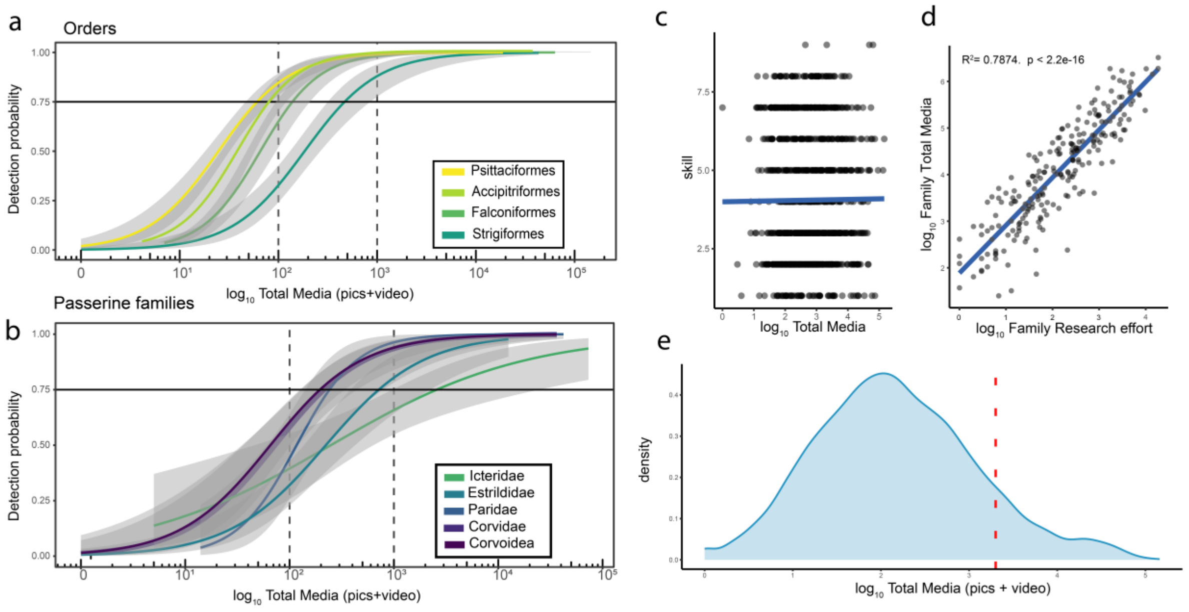

Supplementary figure 4. a, and b, show the correlation between total media and the probability of detecting foot use behavior for different orders (a) and songbird families (b). The curves, derived from a logistic regression, were used to determine the threshold at which foot use behavior could be detected for each clade. Dotted lines show where the lines intersect with 100 and 1000 pictures. Solid lines show the 75% detection probability. **c**, Foot use skill score plotted against log-transformed total media available for 1020 species of birds. Across all birds we found no correlation between skill scores and media available (PGLS, $F_{1,1018,}$= 2.877, p= 0.26). Blue line shows PGLS regression line. **d,** a scatterplot of total media available in the Macaulay Library at the family level plotted against research effort, defined as the total number of papers published on each family ([1]). The solid line indicates a significant correlation between the two (PGLS: $F_{1,202,}$= 103.4, p= >0.001). **e**, A density plot showing the distribution of the total number of media for all species of birds in the Macaulay Library. The red line show where 90 % of the species are found.

Supplementary Table 1: Model parameters for hidden rates analysis for family level foot use behavior.

	-lnL	*AIC*	*AICc*	*# Rate cat*
ER	-141.47	284.94	284.95	1
SYM	-141.47	284.94	284.95	1
ARD	-133.27	270.55	270.60	1
PREC	-127.92	259.84	259.89	2

-lnL=maximum log-likelihood; AIC=Akaike information criterion; AICc=Akaike information criterion corrected for sample size. ER = equal rates, SYM= symmetrical model, all-rates different matrix model, PREC = precursor model. # Rate cat = number of rate categories

Supplementary Table 2 PGLS models parameters and results

Dependent variable	Independent variable	DF	F	P	Lambda (CI)
Skill index	Order	6,1026	3.09	0.008	0.62 (0.45-0.85)
Log_{10} Body Mass	Order (raptors)	3,518	86.9	0.00004	0.99 (0.82-1)
Skill index	Log_{10} Total media	1,1018	2.88	0.26	0.99 (0.85-1)

Supplementary Table 3. Ancestral Diets. Ancestral diet reconstruction. Numbers indicate the ancestral state probability for the most recent common ancestor for each clade (see methods for details).

Clade	*Aquatic pred.*	*Frugivore*	*Granivore*	*Herbivore*	*Invertivore*	*Nectarivore*	*Omnivore*	*Vertivore*
Accipitriformes	0.000	0.000	0.000	0.001	0.000	0.000	0.000	0.999
Strigiformes	0.000	0.000	0.000	0.001	0.000	0.000	0.000	0.999
Falconiformes	0.000	0.000	0.000	0.001	0.000	0.000	0.000	0.999
Psittaciformes	0.003	0.015	0.694	0.063	0.001	0.001	0.210	0.012
Passeriformes	0.000	0.000	0.000	0.000	1.000	0.000	0.000	0.000
Passerida	0.000	0.000	0.003	0.000	0.980	0.000	0.016	0.000
Corvides	0.000	0.000	0.000	0.000	1.000	0.000	0.000	0.000
Piciformes	0.000	0.004	0.000	0.000	0.995	0.000	0.000	0.000
Capitonidae, Semnornithidae, and *Ramphastidae*	0.000	0.998	0.000	0.000	0.000	0.000	0.002	0.000
Sylvioidea	0.00	0.00	0.00	0.00	0.87	0.00	0.13	0.00

Supplementary Table 4. Detection threshold.

	calc. 75 % detection threshold	***Used threshold***
Accipitriformes	123	130
Strigiformes	851	900
Falconiformes	114	120
Psittaciformes	77	85
Piciformes	444	500
Corvidae	446	500
Paridae	794	800
Estrildidae	2344	2400
Icteridae	2754	2800
Corvoidea	338	400

Supplementary Table 5: Model parameters for hidden rates analysis for foot use behavior in parrots

model	*-lnL*	*AIC*	*AICc*	*Rate cat*
ER	-203	408.04	408.01	1
SYM	-194.977	401.95	402.28	1
ARD	-174.122	372.24	373.50	1

-lnL=maximum log-likelihood; AIC=Akaike information criterion; AICc=Akaike information criterion corrected for sample size. ER = equal rates, SYM= symmetrical model, all-rates different matrix model. # Rate cat = number of rate categories

Supplementary Table 6. Pictures information.

ML ID	*Species name*	*English name*	*photographer*	*figure*
ML57273391	*Melanochlora sultanea*	Sultan Tit	Craig Brelsford	fig. 1
ML219550691	*Anodorhynchus hyacinthinus*	Hyacinth Macaw	Nick Athanas	fig. 1
ML204333801	*Elanoides forficatus*	Swallow-tailed Kite	Hal and Kirsten Snyder	fig. 1 and 3
ML126826011	*Athene cunicularia*	Burrowing Owl	Brad Imhoff	fig. 3
ML213918601	*Microhierax caerulescens*	Collared Falconet	Dominic Standing	fig. 3
ML36360411	*Accipiter rufitorques*	Fiji Goshawk	Mat Gilfedder	fig. 3
ML205654991	*Gypohierax angolensis*	Palm-nut Vulture	Frans Vandewalle	fig. 3
ML204478221	*Aegypius monachus*	Cinereous Vulture	Juan Lacruz Martin	fig. 3
ML205223501	*Nestor meridionalis*	New Zealand Kaka	Dave Rintoul	fig. 4
ML66912201	*Psittacara erythrogenys*	Red-masked Parakeet	Walter Oshiro	fig. 4
ML131156891	*Poicephalus cryptoxanthus*	Brown-headed Parrot	Volker Hesse	fig. 4
ML176135231	*Amazona aestiva*	Turquoise-fronted Parrot	Estevão Freitas Santos	fig. 4
ML233718501	*Cacatua sanguinea*	Little Corella	Michael Daley	fig. 4
ML293520261	*Psittacula krameri*	Rose-ringed Parakeet	Dobrin Botev	fig. 4
ML58505951	*Cyanocorax yucatanicus*	Yucatan Jay	Chris Wood	fig. 5
ML449915461	*Vireo flavifrons*	Yellow-throated Vireo	John Garrett	fig. 5
ML141977161	*Mohoua albicilla*	Whitehead	Kuang-Ping Yu	fig. 5
ML409098921	*Cracticus nigrogularis*	Pied Butcherbird	Peter Lowe	fig. 5
ML327037341	*Dicrurus bracteatus*	Spangled Drongo	Ged Tranter	fig. 5
ML231781581	*Lanius minor*	Lesser Gray Shrike	Haldun Savaş	fig. 5
ML229677561	*Dolichonyx oryzivorus*	Bobolink	Court Harding	fig. S3
ML144678231	*Machlolophus aplonotus*	Indian Yellow Tit	Sriram Reddy	fig. S3
ML321218961	*Lonchura castaneothorax*	Chestnut-breasted Munia	Terence Alexander	fig. S3
ML204717411	*Agelaius xanthomus*	Yellow-shouldered Blackbird	Mikko Pyhälä	fig. S3
ML83544481	*Eubucco bourcierii*	Red-headed Barbet	Larry Therrien	fig. S1
ML90815121	*Semnornis ramphastinus*	Toucan Barbet	Eli Gross	fig. S1
ML79312761	*Ramphastos ambiguus*	Yellow-throated Toucan	Krista Kaptein	fig. S1

Supplementary Table 7. List of species and source of foot use in the literature.

Orders	Family	English	Scientific name	Source
Accipitriformes	Accipitridae	Tawny eagle	Aquila rapax	[2]
Accipitriformes	Accipitridae	Verreaux's eagle	Aquila verreauxii	[3]

Accipitriformes	Accipitridae	Double-toothed kite	Harpagus bidentatus	4
Accipitriformes	Accipitridae	Plumbeous kite	Ictinia plumbea	5
Accipitriformes	Accipitridae	Bat hawk	Macheiramphus alcinus	6
Accipitriformes	Accipitridae	The yellow-billed kite	Milvus aegyptius	2,7
Accipitriformes	Cathartidae	Turkey vulture	Cathartes aura	8
Accipitriformes	Pandionidae	Ospreys	Pandion haliaetus	9
Accipitriformes	Sagittariidae	Secretary bird	Sagittarius serpentarius	2
Cariamiformes	Cariamidae	Red-legged seriema	Cariama cristata	10,11
Charadriiformes	Chionidae	Snowy sheathbill	Chionis albus	12
Charadriiformes	Haematopodidae	Pied oystercatcher	Haematopus longirostris	13
Charadriiformes	Stercorariidae	Parasitic jaeger	Stercorarius parasiticus	14
Ciconiiformes	Ciconiidae	Asian openbill stork	Anastomus oscitans	15
Coliiformes	Coliidae	White-backed mousebird	Colius colius	16
Coliiformes	Coliidae	Red-faced mousebird	Colius indicus	16
Coliiformes	Coliidae	Speckled mousebird Birds	Colius striatus	16
Columbiformes	Columbidae	Tooth-billed pigeon	Didunculus strigirostris	17,18
Columbiformes	Columbidae	Marquesan ground dove	Pampusana rubescens	19
Cuculiformes	Cuculidae	Greater coucal	Centropus sinensis	20
Cuculiformes	Cuculidae	Guira cuckoo	Guira guira	21
Falconiformes	Falconidae	Crested caracara	Caracara plancus	22
Galliformes	Megapodiidae	Australian brushturkey	Alectura lathami	21
Gruiformes	Aramidae	Limpkin	Aramus guarauna	23
Gruiformes	Rallidae	Weka	Gallirallus australis	23
Gruiformes	Rallidae	Allen's gallinule	Porphyrio alleni	23
Gruiformes	Rallidae	Purple gallinule	Porphyrio martinica	23
Gruiformes	Rallidae	Western swamphen	Porphyrio porphyrio	23
Passeriformes	Aegithalidae	Long-tailed Tit	Aegithalos caudatus	24
Passeriformes	Aegithalidae	Long-tailed Tit	Aegithalos caudatus	25
Passeriformes	Aegithinidae	Common iora	Aegithina tiphia	7
Passeriformes	Artamidae	Lack-faced woodswallow	Artamus cinereus	26
Passeriformes	Artamidae	White-breasted Woodswallow	Artamus leucorynchus	26
Passeriformes	Artamidae	Great woodswallow	Artamus maximus	23
Passeriformes	Artamidae	Pied butcherbird	Cracticus nigrogularis	27
Passeriformes	Artamidae	Australian magpie	Gymnorhina tibicen	28
Passeriformes	Callaeidae	Huia	Heteralocha acutirostris	29
Passeriformes	Callaeidae	Saddleback	Philesturnus carunculatus	23
Passeriformes	Campephagidae	Bar-bellied Cuckooshrike	Coracina striata	23
Passeriformes	Cardinalidae	Lazuli bunting	Passerina amoena	30
Passeriformes	Cardinalidae	Painted bunting	Passerina ciris	23
Passeriformes	Cinclosomatidae	Chestnut-breasted Quail-thrush	Cinclosoma castaneothorax	23
Passeriformes	Cinclosomatidae	Chestnut Quail-thrush	Cinclosoma castanotum	23
Passeriformes	Cinclosomatidae	Spotted Quail-thrush	Cinclosoma punctatum	23
Passeriformes	Cisticolidae	Common tailorbird	Orthotomus sutorius	31
Passeriformes	Corcoracidae	White-winged Chough	Corcorax melanorhamphos	32
Passeriformes	Corcoracidae	Apostlebird	Struthidea cinerea	33
Passeriformes	Corvidae	Transvolcanic jay	Aphelocoma ultramarina	23,34,35
Passeriformes	Corvidae	White-throated Magpie-Jay	Calocitta formosa	35
Passeriformes	Corvidae	White-necked Raven	Corvus albicollis	35
Passeriformes	Corvidae	Northwestern crow	Corvus caurinus	23
Passeriformes	Corvidae	Carrion crow	Corvus corone	20
Passeriformes	Corvidae	Rook	Corvus frugilegus	35
Passeriformes	Corvidae	Blue jay	Cyanocitta cristata	35
Passeriformes	Corvidae	Steller's jay	Cyanocitta stelleri	36,37
Passeriformes	Corvidae	Purplish jay	Cyanocorax cyanomelas	38
Passeriformes	Corvidae	Green jay	Cyanocorax luxuosus	39
Passeriformes	Corvidae	Brown jay	Cyanocorax morio	40
Passeriformes	Corvidae	Eurasian nutcracker	Nucifraga caryocatactes	41
Passeriformes	Corvidae	Canada jay	Perisoreus canadensis	23
Passeriformes	Corvidae	Canada jay	Perisoreus canadensis	42
Passeriformes	Corvidae	Yellow-billed Magpie	Pica nuttalli	43
Passeriformes	Corvidae	Red billed cough	Pyrrhocorax pyrrhocorax	44
Passeriformes	Corvidae	Stresemann's bush-crow	Zavattariornis stresemanni	23
Passeriformes	Dicruridae	Fork-tailed drongo	Dicrurus adsimilis	45
Passeriformes	Dicruridae	Shining drongo	Dicrurus atripennis	23
Passeriformes	Dicruridae	White-bellied drongo	Dicrurus caerulescens	28

Passeriformes	Dicruridae	Tablas drongo	Dicrurus menagei	[23]
Passeriformes	Dicruridae	Velvet-mantled Drongo	Dicrurus modestus	[23]
Passeriformes	Dicruridae	Western Square-tailed Drongo	Dicrurus occidentalis	[23]
Passeriformes	Dicruridae	Greater Racket-tailed	Dicrurus paradiseus	[25]
Passeriformes	Dicruridae	Sharpe's drongo	Dicrurus sharpei	[23]
Passeriformes	Estrildidae	Red-headed Parrotfinch	Erythrura cyaneovirens	[23]
Passeriformes	Estrildidae	Gouldian finch	Erythrura gouldiae	[23]
Passeriformes	Estrildidae	Pink-billed Parrotfinch	Erythrura kleinschmidti	[23]
Passeriformes	Estrildidae	Royal parrotfinch	Erythrura regia	[23]
Passeriformes	Estrildidae	Common waxbill	Estrilda astrild	[23,46]
Passeriformes	Estrildidae	Black-crowned Waxbill	Estrilda nonnula	[23,47]
Passeriformes	Estrildidae	Black-tailed Waxbill	Estrilda perreini	[23]
Passeriformes	Estrildidae	Crimson-rumped Waxbill	Estrilda rhodopyga	[23]
Passeriformes	Estrildidae	Black-rumped Waxbill	Estrilda troglodytes	[48]
Passeriformes	Estrildidae	Indian silverbill	Euodice malabarica	[46]
Passeriformes	Estrildidae	Lavender waxbill	Glaucestrilda caerulescens	[48,49]
Passeriformes	Estrildidae	Gray-headed Munia	Lonchura caniceps	[50]
Passeriformes	Estrildidae	Magpie munia	Lonchura fringilloides	[50]
Passeriformes	Estrildidae	Black munia	Lonchura stygia	[50]
Passeriformes	Estrildidae	White-collared oliveback	Nesocharis ansorgei	[51]
Passeriformes	Estrildidae	Java sparrow	Padda oryzivora	[23]
Passeriformes	Estrildidae	Bronze mannikin	Spermestes cucullata	[46]
Passeriformes	Estrildidae	Western bluebill	Spermophaga haematina	[52]
Passeriformes	Estrildidae	Red-eared Firetail	Stagonopleura oculata	[23,26]
Passeriformes	Estrildidae	Violet-eared Waxbill	Uraeginthus granatinus	[23]
Passeriformes	Falcunculidae	Crested shrike-tit	Falcunculus frontatus	[53]
Passeriformes	Fringillidae	European goldfinch	Carduelis carduelis	[54]
Passeriformes	Fringillidae	European greenfinch	Carduelis chloris	[23,54]
Passeriformes	Fringillidae	Citril finch	Carduelis citrinella	[23]
Passeriformes	Fringillidae	Common redpoll	Carduelis flammea	[54]
Passeriformes	Fringillidae	Hoary redpoll	Carduelis hornemanni	[23]
Passeriformes	Fringillidae	Eurasian siskin	Carduelis spinus	[54]
Passeriformes	Fringillidae	American goldfinch	Carduelis tristis	[23,55]
Passeriformes	Fringillidae	Common chaffinch	Fringilla coelebs	[56,57]
Passeriformes	Fringillidae	Oahu amakihi	Hemignathus flava	[23]
Passeriformes	Fringillidae	Akiapolaau	Hemignathus munroi	[23]
Passeriformes	Fringillidae	Kauai amakihi	Hemignathus stejnegeri	[23]
Passeriformes	Fringillidae	Hawaii amakihi	Hemignathus virens	[23]
Passeriformes	Fringillidae	Laysan ‘apapane	Himatione fraithii	[58]
Passeriformes	Fringillidae	Apapane	Himatione sanguinea	[23]
Passeriformes	Fringillidae	Twite	Linaria flavirostris	[57]
Passeriformes	Fringillidae	Red crossbill	Loxia curvirostra	[23,54]
Passeriformes	Fringillidae	White-winged Crossbill	Loxia leucoptera	[59]
Passeriformes	Fringillidae	Parrot crossbill	Loxia pytyopsittacus	[23]
Passeriformes	Fringillidae	Maui alauahio	Paroreomyza montana	[23]
Passeriformes	Fringillidae	Maui parrotbill	Pseudonestor xanthophrys	[23]
Passeriformes	Fringillidae	Island canary	Serinus canaria	[23]
Passeriformes	Fringillidae	Streaky-headed Seedeater	Serinus gularis	[23]
Passeriformes	Fringillidae	European serin	Serinus serinus	[23]
Passeriformes	Fringillidae	Pine siskin	Spinus pinus	[35]
Passeriformes	Furnariidae	Pink-legged Graveteiro	Acrobatornis fonsecai	[60]
Passeriformes	Furnariidae	Olive-backed foliage-gleaner	Automolus infuscatus	[61]
Passeriformes	Furnariidae	Buff-throated Foliage-gleaner	Automolus ochrolaemus	[62]
Passeriformes	Furnariidae	Rufous-fronted thornbird	Phacellodomus rufifrons	[62]
Passeriformes	Furnariidae	Rufous-rumped foliage-gleaner	Philydor erythrocercum	[63]
Passeriformes	Furnariidae	Russet-mantled Foliage-gleaner	Syndactyla dimidiata	[64]
Passeriformes	Icteridae	Bay-winged Cowbird	Agelaioides badius	[23]
Passeriformes	Icteridae	Yellow-shouldered Blackbird	Agelaius xanthomus	[23]
Passeriformes	Icteridae	Pale-eyed Blackbird	Agelaius xanthophthalmus	[65]
Passeriformes	Icteridae	Yellow-billed Cacique	Amblycercus holosericeus	[37]
Passeriformes	Icteridae	Scarlet-rumped cacique	Cacicus uropygialis	[66]
Passeriformes	Icteridae	Melodious blackbird	Dives dives	[40]
Passeriformes	Icteridae	Bobolink	Dolichonyx oryzivorus	[67]
Passeriformes	Icteridae	Rusty blackbird	Euphagus carolinus	[23]
Passeriformes	Icteridae	Brewer's blackbird	Euphagus cyanocephalus	[23,68]
Passeriformes	Icteridae	Baltimore oriole	Icterus galbula	[23,69]

Passeriformes	Icteridae	Baltimore oriole	Icterus galbula	69
Passeriformes	Icteridae	Montserrat oriole	Icterus oberi	23
Passeriformes	Icteridae	Orchard oriole	Icterus spurius	23,70
Passeriformes	Icteridae	Brown-headed Cowbird	Molothrus ater	59
Passeriformes	Icteridae	Boat-tailed Grackle	Quiscalus major	23
Passeriformes	Icteridae	Great-tailed Grackle	Quiscalus mexicanus	35
Passeriformes	Icteridae	Common grackle	Quiscalus quiscula	35,71
Passeriformes	Icteriidae	Yellow-breasted Chat	Icteria virens	23,72
Passeriformes	Laniidae	Commonfiscal	Lanius collaris	73
Passeriformes	Laniidae	Fiscal shrike	Lanius collaris	2
Passeriformes	Laniidae	Red-backed Shrike -	Lanius collurio	74
Passeriformes	Laniidae	Great grey shrike	Lanius excubitor	23,75
Passeriformes	Laniidae	Loggerhead shrikes	Lanius ludovicianus	76
Passeriformes	Laniidae	Lesser gray shrike	Lanius minor	77
Passeriformes	Leiothrichidae	Blue-winged minla	Actinodura cyanouroptera	7
Passeriformes	Leiothrichidae	Striated babbler	Argya earlei	7
Passeriformes	Leiothrichidae	Black-headed sibia	Heterophasia desgodinsi	7
Passeriformes	Leiothrichidae	Silver-eared mesia	Leiothrix argentauris	7,78
Passeriformes	Leiothrichidae	Red-billed Leiothrix	Leiothrix lutea	28
Passeriformes	Leiothrichidae	White-throated Laughingthrush	Pterorhinus albogularis	45
Passeriformes	Leiotrichidae	Chinese hwamei	Garrulax canorus	28
Passeriformes	Leiotrichidae	White-crested Laughingthrush	Garrulax leucolophus	28
Passeriformes	Leiotrichidae	Striated laughingthrush	Grammatoptila striata	28
Passeriformes	Leiotrichidae	Yellow-billed Babbler	Turdoides affinis	45
Passeriformes	Leiotrichidae	Arrow-marked Babbler	Turdoides jardineii	23
Passeriformes	Locustellidae	New zealand fernbird	Poodytes punctatus	79
Passeriformes	Malaconotidae	Ethiopian boubou	Laniarius aethiopicus	23
Passeriformes	Malaconotidae	Grey-headed bushshrike	Malaconotus blanchoti	23
Passeriformes	Malaconotidae	Fiery-breasted Bushshrike	Malaconotus cruentus	23
Passeriformes	Malaconotidae	Brubru	Nilaus afer	23
Passeriformes	Malacotonidae	The brown-crowned tchagra	Tchagra australis	2
Passeriformes	Meliphagidae	Long-billed honeyeater	Melilestes megarhynchus	80
Passeriformes	Meliphagidae	Mimic meliohaga	Meliphaga analoga	80
Passeriformes	Meliphagidae	Puff-backed honeyeater	Meliphaga aruensis	80
Passeriformes	Meliphagidae	Mottle-breasted honeyeater	Meliphaga mimikae	80
Passeriformes	Meliphagidae	Helmeted friarbird	Philemon buceroides	80
Passeriformes	Meliphagidae	Striped honeyeater	Plectorhyncha lanceolata	23
Passeriformes	Meliphagidae	Tawny-breasted Honeyeater	Xanthotis flaviventer	80
Passeriformes	Meliphagidae	Spotted honeyeater	Xanthotis polygrammus	80
Passeriformes	Menuridae	Superb lyrebird	Menura novaehollandie	81
Passeriformes	Mimidae	California thrasher	Toxostoma redivivum	35
Passeriformes	Mohouidae	Yellowhead	Mohoua ochrocephala	23
Passeriformes	Monarchidae	Elepaio	Chasiempis sandwichensis	23
Passeriformes	Monarchidae	Black-naped Monarch	Hypothymis azurea	23
Passeriformes	Monarchidae	Black-faced Monarch	Monarcha melanopsis	82
Passeriformes	Motacillidae	White wagtail	Motacilla alba	23
Passeriformes	Nectariniidae	Seychelles sunbird	Cinnyris dussumieri	83
Passeriformes	Neosittidae	Varied sittella	Daphoenositta chrysoptera	23
Passeriformes	Panuridae	Bearded reedling	Panurus biarmicus	84
Passeriformes	Paradisaeidae	Magnificent Bird of Paradise	Cicinnurus magnificus	80,85
Passeriformes	Paradisaeidae	Crinkle-collared manucode	Manucodia chalybatus	80
Passeriformes	Paradisaeidae	Raggiana bird-of-paradise	Paradisaea raggiana	80,85
Passeriformes	Paradisaeidae	Red bird-of-paradise	Paradisaea rubra	86
Passeriformes	Paradisaeidae	Wahnes's parotia	Parotia wahnesi	87
Passeriformes	Paradisaeidae	Magnificent riflebird	Ptiloris magnificus	80,85
Passeriformes	Paradisaeidae	Twelve-wired bird-of-paradise	Seleucidis melanoleucus	88
Passeriformes	Paradoxornithidae	Yellow-eyed Babbler	Chrysomma sinense	28
Passeriformes	Paradoxornithidae	The yellow-eyed babbler	Chrysomma sinense	89
Passeriformes	Paradoxornithidae	Gray-headed Parrotbill	Paradoxornis gularis	23
Passeriformes	Paridae	Oak titmouse	Baeolophus inornatus	90

Passeriformes	Paridae	Fire-capped Tit	Cephalopyrus flammiceps	[23]
Passeriformes	Paridae	Gray tit	Parus afer	[91]
Passeriformes	Paridae	Black-capped Chickadee	Parus atricapillus	[35,92]
Passeriformes	Paridae	Eurasian blue tit	Parus caeruleus	[91]
Passeriformes	Paridae	Carolina chickadee	Parus carolinensis	[92]
Passeriformes	Paridae	Mountain chickadee	Parus gambeli	[35]
Passeriformes	Paridae	Sombre tit	Parus lugubris	[93]
Passeriformes	Paridae	Japanese tit	Parus major	[91]
Passeriformes	Paridae	Carolina chickadee	Poecile carolinensis	[92]
Passeriformes	Parulidae	Yellow-rumped Warbler	Dendroica coronata	[94]
Passeriformes	Parulidae	Worm-eating Warbler	Helmitheros vermivorum	[23]
Passeriformes	Passerellidae	Grassland sparrow	Ammodramus humeralis	[23]
Passeriformes	Passeridae	House sparrow	Passer domesticus	[95]
Passeriformes	Platysteiridae	Chinspot batis	Batis molitor	[96]
Passeriformes	Ploceidae	Aldabra fody	Foudia aldabrana	[97]
Passeriformes	Ploceidae	Comoros fody	Foudia eminentissima	[98]
Passeriformes	Ploceidae	Red-headed Malimbe	Malimbus rubricollis	[23]
Passeriformes	Ploceidae	Baya weaver	Ploceus philippinus	[99]
Passeriformes	Pomatostomidae	Papuan babbler	Pomatostomus isidorei	[23]
Passeriformes	Psophodidae	Eastern whipbird	Psophodes olivaceus	[23]
Passeriformes	Pycnonotidae	Yellow-eared Bulbul	Pycnonotus penicillatus	[100]
Passeriformes	Regulidae	Ruby-crowned kinglet	Regulus calendula	[101]
Passeriformes	Regulidae	The tenerife kinglet	Regulus teneriffae	[102]
Passeriformes	Remizidae	Southern penduline-tit	Anthoscopus minutus	[23,103]
Passeriformes	Remizidae	Verdin	Auriparus flaviceps	[104]
Passeriformes	Remizidae	Eurasian penduline-tit	Remiz pendulinus	[23]
Passeriformes	Rhipiduridae	New zealand fantail	Rhipidura fuliginosa	[82]
Passeriformes	Rhipiduridae	Willie-wagtail	Rhipidura leucophrys	[23,84]
Passeriformes	Rhipiduridae	Rufous fantail	Rhipidura rufifrons	[23]
Passeriformes	Sittidae	Snowy-browed Nuthatch	Sitta villosa	[23]
Passeriformes	Sturnidae	Brahminy starling	Sturnus pagodarum	[105]
Passeriformes	Sylviidae	Wrentit	Chamaea fasciata	[23]
Passeriformes	Sylviidae	Wrentit	Chamaea fasciata	[106]
Passeriformes	Thraupidae	Woodpecker finch	Camarhynchus pallidus	[107]
Passeriformes	Thraupidae	Small tree-finch	Camarhynchus parvulus	[108]
Passeriformes	Thraupidae	Large Tree-finch	Camarhynchus psittacula	[108]
Passeriformes	Thraupidae	Green warbler finch	Certhidea olivacea	[108]
Passeriformes	Thraupidae	Lesser antillean bullfinch	Loxigilla noctis	[23]
Passeriformes	Thraupidae	Puerto rican bullfinch	Loxigilla portoricensis	[23]
Passeriformes	Thraupidae	Fawn-breasted tanager	Pipraeidea melanonota	[109]
Passeriformes	Thraupidae	Temminck's seedeater	Sporophila falcirostris	[110]
Passeriformes	Thraupidae	Buffy-fronted Seedeater	Sporophila frontalis	[110]
Passeriformes	Thraupidae	Yellow-faced Grassquit	Tiaris olivaceus	[111]
Passeriformes	Timaliidae	White-browed Scimitar-Babbler	Pomatorhinus schisticeps	[28]
Passeriformes	Turdidae	Wood thrush	Hylocichla mustelina	[112]
Passeriformes	Turdidae	Townsend's solitaire	Myadestes townsendi	[23]
Passeriformes	Tyrannidae	Pacific-slope Flycatcher	Empidonax difficilis	[113]
Passeriformes	Tyrannidae	Willow flycatcher	Empidonax traillii	[68,114]
Passeriformes	Tyrannidae	Streaked flycatcher	Myiodynastes maculatus	[115]
Passeriformes	Tyrannidae	White-rumped Monjita	Xolmis velatus	[116]
Passeriformes	Vangidae	Red-shouldered Vanga	Calicalicus rufocarpalis	[23]
Passeriformes	Vangidae	Helmet vanga	Euryceros prevostii	[117]
Passeriformes	Vangidae	Sickle-billed Vanga	Falculea palliata	[23]
Passeriformes	Vangidae	Tylas vanga	Tylas eduardi	[23]
Passeriformes	Viduidae	Pin-tailed Whydah	Vidua macroura	[23]
Passeriformes	Vireonidae	Rufous-browed Peppershrike	Cyclarhis gujanensis	[37]
Passeriformes	Vireonidae	Gray-eyed Greenlet	Hylophilus amaurocephalus	[23]
Passeriformes	Vireonidae	Bells vireo	Vireo bellii	[118]
Passeriformes	Vireonidae	Noronha vireo	Vireo gracilirostris	[23]
Passeriformes	Vireonidae	White-eyed Vireo	Vireo griseus	[23]
Passeriformes	Vireonidae	Plumbeous vireo	Vireo plumbeus	[23]
Passeriformes	Vireonidae	Blue-headed Vireo	Vireo solitarius	[23,119]
Passeriformes	Vireonidae	Gray vireo	Vireo vicinior	[23]
Passeriformes	Vireonidae	Chestnut-sided Shrike-Vireo	Vireolanius melitophrys	[23]
Passeriformes	Zosteropidae	Mascarene Grey White-eye	Zosterops borbonica	[120]
Passeriformes	Zosteropidae	Réunion olive white-eye	Zosterops olivacea	[120]
Passeriformes	Zosteropidae	Swinhoe's white-eye	Zosterops simplex	[7]

Pelecaniformes	Ardeidae	Grey heron	Ardea cinerea	121
Pelecaniformes	Ardeidae	Blue heron	Ardea herodias	122
Pelecaniformes	Ardeidae	Green-backed Heron	Ardeola striata	123
Pelecaniformes	Ardeidae	Pacific reef heron	Egretta sacra	124
Pelecaniformes	Threskiornithidae	Australian white ibis	Threskiornis molucca	125
Piciformes	Capitonidae	Red-headed Barbet	Eubucco bourcierii	23
Piciformes	Picidae	Golden-fronted woodpecker	Melanerpes aurifrons	126
Piciformes	Ramphastidae	Plate-billed Mountain-Toucan	Andigena laminirostris	23
Piciformes	Ramphastidae	Chestnut-mandibled toucan	Ramphastos ambiguus	66
Piciformes	Semnornithidae	Prong-billed Barbet	Semnornis frantzii	23
Piciformes	Semnornithidae	Toucan barbet	Semnornis ramphastinus	23
Psittaciformes	Cacatuidae	Tanimbar corella	Cacatua goffiniana	127
Psittaciformes	Cacatuidae	Salmon-crested cockatoo	Cacatua moluccensis	39
Psittaciformes	Cacatuidae	Palm cockatoo	Probosciger aterrimus	128
Psittaciformes	Psittacidae	Hyacinth macaws	Anodorhynchus hyacinthinus	129
Psittaciformes	Psittacidae	Cape parrot	Poicephalus robustus	130
Psittaciformes	Strigopidae	Kakapo	Strigops habroptilus	131
Psittasiformes	Psittaculidae	Australian ringneck	Barnardius zonarius	27,132
Strigiformes	Strigidae	Long-eared owl	Asio otus	133
Strigiformes	Strigidae	Akun Eagle-owl	Bubo leucostictus	23
Strigiformes	Strigidae	Whiskered Screech-owl	Megascops trichopsis	23
Strigiformes	Tytonidae	Barn owl	Tyto alba	134
Suliformes	Phalacrocoracidae	Reed cormorant	Microcarbo africanus	135
Suliformes	Phalacrocoracidae	Little pied cormorant	Microcarbo melanoleucos	136
Suliformes	Phalacrocoracidae	Little cormorant	Microcarbo niger	136

Supplementary references

1. Ducatez, S., Sol, D., Sayol, F. & Lefebvre, L. Behavioural plasticity is associated with reduced extinction risk in birds. *Nat Ecol Evol* **4**, 788–793 (2020).
2. Van Someren, V. G. L. Days with Birds: Studies of Habits of some East Africa birds. in *Fieldiana Zoology* vol. v.38 (1956) ([Chicago] Chicago Natural History Museum [1945?-, 1956).
3. Mundy, P. J., Robertson, A. S., Komen, J. & O'connor, T. G. Attacks by black eagles on vultures. *Raptor research* **20**, 61–64 (1986).
4. Baker, A. J., Whitacre, D. F. (David F., Aguirre-Barrera, O. A., López Ávila, Juventino. & White, C. M. Observation of a Double-toothed Kite (Harpagus bidentatus) hawking bats. *The journal of raptor research* **33**, 343–344 (1999).
5. Seavy, N. E., Schulze, M. D., Whitacre, D. F. (David F. & Vasquez, M. A. Breeding Biology and Behavior of the Plumbeous Kite. *The Wilson bulletin* **110**, 77–85 (1998).
6. Thomsett, S. Some observations on the bat hawk Macheiramphus alcinus. *Scopus* **5**, 56–56 (1981).

7. Finn, F. Stray Notes on Indian Birds. in *The Avicultural magazine* vol. [v.15]=new ser.:v.7 (1908-1909) (Ascot, Berkshire, etc, Avicultural Society, etc, 1908).

8. Kempton, R. M. Notes on the Home Life of the Turkey Vulture. *The Wilson bulletin* **39**, 142–145 (1927).

9. Sustaita, D., Gloumakov, Y., Tsang, L. R. & Dollar, A. M. Behavioral correlates of semi-zygodactyly in Ospreys (Pandion haliaetus) based on analysis of internet images. *PeerJ* **7**, e6243 (2019).

10. Finn, Frank. *Talks about birds*. (A. and C. Black, 1911).

11. Oswald, T., Curtice, B., Bolander, M. & Lopez, C. Observation of Claw Use and Feeding Behavior of the Red-Legged Seriema and Its Implication for Claw Use in Deinonychosaurs. *jana* **50**, 17–21 (2023).

12. Finn, F. Method of Feeding in hte Sheatbill. *The Avicultural magazine* **[v.34]=ser.4:v.6 (1928)**, 98 (1928).

13. Jones, Thomas Rymer. *The natural history of birds; a popular introduction to ornithology*. (London, Society for Promoting Christian Knowledge, 1867).

14. Pruett-Jones, S. G. Team-Hunting and Food Sharing in Parasitic Jaegers. *The Wilson Bulletin* **92**, 524–526 (1980).

15. Bingham, C. T. Anastomus oscitans. in *Stray feathers. Journal of ornithology for India and its dependencies* vol. v. 4 (1876) (Calcutta, s.n, 1872-1899, 1876).

16. Rowan, M. A study of the colies of southern Africa. *Ostrich* **38**, 63–115 (1967).

17. Gifford, E. W. The Gray-Hooded Quail Dove (Gallicolumba rubescens) of the Marquesas Islands, in Captivity. *The Auk* **42**, 388–396 (1925).

18. Collar, N. J. Natural history and conservation biology of the tooth-billed pigeon (Didunculus strigirostris): a review. *Pac. Conserv. Biol.* **21**, 186–199 (2015).

19. Smith, G. The use of the foot in feeding, with especial reference to parrots. *Avicult. Mag* **77**, 93–100 (1971).

20. Finn, F. *Birds of Our Country: Their eggs, nests, life, haunts and identification*. vol. 1 (Hutchinson, 1922).

21. Finn, F. *Bird behaviour, psychical and physiological;* (London, Hutchinson, 1919).

22. Kilham, L. Courtship of common caracaras in Costa Rica. *Raptor research* **13**, 17–19 (1979).

23. *Birds of the World*. (Cornell Laboratory of Ornithology, 2022).

24. Harrap, S. Long-tailed Tit (Aegithalos caudatus), version 1.0. *Birds of the World* https://birdsoftheworld.org/bow/species/lottit1/cur/introduction (2020).

25. Simmons, K. Some behaviour characters of the babblers (Timaliidae). *Avicultural Magazine* **69**, 183–193 (1963).

26. Immelmann, K. Beobachtungen an Schwalbenstaren. *J Ornithol* **107**, 37–69 (1966).

27. Milligan, Alex. Wm. Notes on a Trip to the Yandanooka District, Western Australia. *Emu - Austral Ornithology* **4**, 151–157 (1905).

28. Kramer, G. Bewegungsstudien an Vögeln des Berliner Zoologischen Gartens. *Journal für Ornithologie* **78**, 257–268 (1930).

29. Buller, S. W. L. *A History of the Birds of New Zealand*. (The Author, 1888).

30. Miller, A. H. Foraging Dexterity of a Lazuli Bunting. *The Condor* **41**, 255–256 (1939).

31. Touche, J. D. L. & Rickett, C. B. II.-Further Notes on the Nesting o j Birds in the Province of Foibkien, S.E. China. *Ibis* **47**, 25–67 (1905).

32. Hobbs, J. N. Use of tools by the White-winged Chough. *EMU* **71**, 84–85 (1971).

33. Baldwin, M. Studies of the apostle bird at Inverell part 1: General behaviour. *The Sunbird: Journal of the Queensland Ornithological Society* **5**, 77–88 (1974).

34. Swarth, H. S. *Birds of the Huachuca Mountains, Arizona*. (The Club, 1900).

35. Clark, G. A. Holding Food with the Feet in Passerines. *Bird-Banding* **44**, 91–99 (1973).

36. Risdon, D. H. S. The breeding of Steller's Jay (Cyanocitta stelleri) at Dudley Zoo. in *The Avicultural magazine* vol. v.66 (1960) (Ascot, Berkshire, etc, Avicultural Society, etc, 1960).

37. Skutch, A. F. *Life histories of Central American highland birds*. https://www.biodiversitylibrary.org/item/260537 (1967).

38. Wetmore, A. *Observations on the birds of Argentina, Paraguay, Uruguay, and Chile*. (US Government Printing Office, 1926).

39. Boswall, J. Tool-using and related behaviour in birds: More notes. *Aviculture Magazine* **89**, 94–108 (1983).

40. Skutch, A. Life histories of Central American birds, vol 1 (Pacific Coast Avifauna 31). *Cooper Ornithological Society, Berkeley* (1954).

41. Witherby, H. F. *et al. Handbook of British birds*. (H.F. & G. Witherby ltd., 1943).

42. Ouellet, H. Further observations on the food and predatory habits of the gray jay. *Can. J. Zool.* **48**, 327–330 (1970).

43. Linsdale, J. The natural history of magpies. Pacific. *Coast Avifauna* **25**, (1937).

44. Hunt, John. *British ornithology : containing portraits of all the British birds including those of foreign origin which have become domesticated*. vol. v.2 (Norwich, Bacon, 1815-22, 1815).

45. Ali, S. The Book of Indian Birds. 158+ li, Bombay Natural History. *Society, Bombay* (1961).

46. Immelmann, K. & Immelmann, K. *Periodische Vorgänge in der Fortpflanzung tierischer Organismen*. (Springer, 1967).

47. Goodwin, D. Some Notes on Black-capped Waxbills. *The Avicultural magazine* **v.69 (1963)**, (1963).

48. Harrison, C. J. O. An Ethological Comparison of Some Waxbills (estrildini), and Its Relevence to Their Taxonomy. *Proceedings of the Zoological Society of London* **139**, 261–282 (1962).

49. Hinze, I. Waxbills And Their Allies: The Lavender Waxbill. *The Avicultural magazine* **106**, 80–85 (2000).

50. Restall, R. *Munias and mannikins.* (Pica Press, 1996).

51. Clement, P. *Finches and sparrows*. (Bloomsbury Publishing, 1999).

52. Harrison, C. J. O. Some notes on the behaviour of the Red-breasted Blue-bill (Spermophaga haematina). *Aviculture Magazine* **72**, 16–17 (1966).

53. Noske, R. A. Left-footedness and tool-using in the varied sittella Daphoenositta chrysoptera and crested shrike-tit Falcunculus frantatus. *Corella* **9**, 63–64 (1985).

54. Newton, I. The Adaptive Radiation and Feeding Ecology of Some British Finches. *Ibis* **109**, 33–96 (1967).

55. Coutlee, E. L. Maintenance Behavior of the American Goldfinch. *The Wilson Bulletin* **75**, 342–357 (1963).

56. Marler, P. Behaviour of the Chaffinch Fringilla Coelebs. *Behaviour. Supplement* III–184 (1956).

57. Kear, J. Food Selection in Finches with Special Reference to Interspecific Differences. *Proceedings of the Zoological Society of London* **138**, 163–204 (1962).

58. Fisher, Walter K. (Walter Kenrick). Notes on the Birds Peculiar to Laysan Island, Hawaiian Group. *The Auk* **20**, 384–397 (1903).

59. Bent, A. C. & Austin, O. L. Life Histories of North American Cardinals, Grosbeaks, Buntings, Towhees, Finches, Sparrows, and Allies. (1968).

60. Pacheco, J. F., Whitney, B. M. & Gonzaga, L. P. A new genus and species of furnariid (Aves: furnariidae) from the cocoa-growing region of southeastern Bahia, Brazil. *Wilson Bulletin* **108**, 397–434 (1996).

61. Zimmer, K. J. Species Limits in Olive-Backed Foliage-Gleaners (Automolus: Furnariidae). *The Wilson bulletin* **114**, 20–37 (2002).

62. Skutch, A. F. A Study of the Rufous-Fronted Thornbird and Associated Birds: Part 1. Life History of the Rufous-Fronted Thornbird. *The Wilson Bulletin* **81**, 5–43 (1969).

63. Rosenberg, K. V. Ecology of Dead-Leaf Foraging Specialists and Their Contribution to Amazonian Bird Diversity. *Ornithological Monographs* 673–700 (1997) doi:10.2307/40157560.

64. Robbins, M. B. & Zimmer, K. J. Taxonomy, vocalisations and natural history of Philydor dimidiatum (Furnariidae), with comments on the systematics of Syndactyla and Simoxenops. *Bulletin of the British Ornithologists' Club* **125**, 212–228 (2005).

65. Orians, G. H. & Orians, E. N. Observations of the Pale-Eyed Blackbird in Southeastern Peru. *The Condor* **102**, 956–958 (2000).

66. Skutch, Alexander F. (Alexander Frank) & Club, N. O. *Studies of tropical American birds*. (Cambridge, Mass, The Club, 1972, 1972).

67. Gosse, Philip Henry, Deane, R. & Hill, Richard. *The birds of Jamaica*. (London, J. Van Voorst, 1847).

68. La Rivers, I. The Mormon Cricket as Food for Birds. *The Condor* **43**, 65–69 (1941).

69. Wellman, G. B. Baltimore Oriole Feeding on Larvae of Needle Miner. *The Auk* **45**, 507–507 (1928).

70. Lohrer, F. E. Orchard Oriole holding food with the feet. *Florida Field Naturalist* **5**, 51 (1977).

71. Roberts, T. S. *The birds of Minnesota. Vol. II.* (Univ. of Minnesota Press, 1932).

72. Ficken, M. S. Agonistic Behavior and Territory in the American Redstart. *The Auk* **79**, 607–632 (1962).

73. Cooper, J. The breeding of the Fiscal Shrike in southern Africa. *Ostrich* **42**, 166–174 (1971).

74. Ash, J. Observations on a decreasing population of Red-backed Shrikes. *Brit. Birds* **63**, 225–239 (1970).

75. Cade, T. J. *Ecological and behavioral aspects of predation by the Northern Shrike*. (1967).

76. Miller, A. H. *Systematic revision and natural history of the American shrikes (Lanius)*. vol. 38 (University of California Press, 1931).

77. Ullrich, B. *Untersuchungen zur Ethologie und Ökologie des Rotkopfwürgers (Lanius senator) in Südwestdeutschland im Vergleich zu Raubwürger (L. excubitor), Schwarzstirnwürger (L. minor) und Neuntöter (L. collurio)*. (1971).

78. Gibson, L. The silver eared mesia. in *The Avicultural magazine* vol. v.97 (1991) (Ascot, Berkshire, etc, Avicultural Society, etc, 1991).

79. Best, H. A. Food and foraging behaviour of the Snares fernbird. *New Zealand Journal of Zoology* **6**, 481–488 (1979).

80. Brown, E. D. & Hopkins, M. J. G. Tests of disperser specificity between frugivorous birds and rainforest fruits in New Guinea. *EMU* **102**, 137–146 (2002).

81. Austin, V. I., Welbergen, J. A., Maisey, A. C., Lindsay, M. G. & Dalziell, A. H. Destruction of a conspecific nest by a female Superb Lyrebird: evidence for reproductive suppression in a bird with female-only parental care. *Behaviour* **156**, 1459–1469 (2019).

82. Harrison, C. J. O. Helpers at the nest in Australian passerine birds (Results of the Harold Hall Australian Expedition, No.21). *EMU* **69**, 30–40 (1969).

83. Greig-Smith, P. W. Foraging, Seasonality and Nesting of Seychelles Sunbirds Nectarinia Dussumieri. *Ibis* **122**, 307–321 (1980).

84. Koenig, O. Okologie und Verhalten der Vogel des Neusiedlersee-Schilf- giirtels. *J. f. Ornithol* **93**, 207–289 (1952).

85. Beehler, B. M. & Dumbacher, J. P. More Examples of Fruiting Trees Visited Predominantly by Birds of Paradise. *EMU* **96**, 81–88 (1996).

86. Frith, C. B. Displays of the Red Bird-of-Paradise Paradisaea Rubra and Their Significance, with a Discussion on Displays and Systematics of Other Paradisaeidae. *Emu - Austral Ornithology* **76**, 69–78 (1976).

87. Frith, C. B. & Frith, D. W. Leaf-eating by birds-of-paradise and bowerbird. *The Sunbird: Journal of the Queensland Ornithological Society* **10**, 21–23 (1979).

88. Rand, A. L. & Gilliard, E. T. *Handbook of new guinea birds*. (Weidenfeld & Nicolson, 1967).

89. Harper, E. W. The Yellow eyed Babbler. in *The Avicultural magazine* vol. v.8 (1901-1902) (Ascot, Berkshire, etc, Avicultural Society, etc, 1901).

90. Root, R. B. The Niche Exploitation Pattern of the Blue-Gray Gnatcatcher. *Ecological Monographs* **37**, 317–350 (1967).

91. Yince, M. A. Use of the Feet in Feeding by the Great Tit Parus Major. *Ibis* **106**, 508–529 (1964).

92. Brewer, Richard. Comparative Notes on the Life History of the Carolina Chickadee. *The Wilson bulletin* **73**, 348–373 (1961).

93. Löhrl, H. Zur Biologie der Trauermeise(Parus lugubris). *J Ornithol* **107**, 167–186 (1966).

94. Conway, W. C., Smith, L. M. & Bergan, J. F. Avian Use of Chinese Tallow Seeds in Coastal Texas. *The Southwestern Naturalist* **47**, 550–556 (2002).

95. Summers-Smith, D. The House Sparrow. London. (1963).

96. Harris, T. *Shrikes and Bush-shrikes: Including Wood-shrikes, Helmet-shrikes, Shrike Flycatchers, Philentomas, Batises and Wattle-eyes*. (A&C Black, 2010).

97. Frith, C. B. A Twelve-Month Field Study of the Aldabran Fody Foudia Eminentissima Aldabrana. *Ibis* **118**, 155–178 (1976).

98. Cheke, A. S. (Anthony S.) & Diamond, A. W. BIRDS ON MOHELI AND GRANDE COMORE COMORO ISLANDS IN FEBRUARY 1975. *Bulletin of the British Ornithologists' Club* **106**, 138–148 (1986).

99. Das, S., Bhowmick, M., Chattopadhyay, S. K. & Basak, S. Application of biomimicry in textiles. *Current Science* **109**, 893–901 (2015).

100. Chandrasiri, P. H. S. P. & Mahaulpatha, W. a. D. Frugivory of Yellow-eared Bulbul (Pycnonotus penicillatus) and Seasonal Variation of Fruiting Phenology in Tropical Montane Cloud Forests of Horton Plains National Park, Sri Lanka. (2019).

101. Ross, R. C. Occurrence and Behavior of Certain Shorebirds in Southern California. *The Condor* **26**, 90–92 (1924).

102. Löhrl, H., Thaler, E. & Christie, A. D. Status and behaviour of the Tenerife Goldcrest. *Brit. Birds* **89**, 379-386. (1996).

103. Skead, C. A study of the Cape Penduline Tit Anthoscopus minutus minutus (Shaw & Nodder). *Ostrich* **30**, 274–288 (1959).

104. Taylor, W. K. A Breeding Biology Study of the Verdin, Auriparus flaviceps (Sundevall) in Arizona. *The American Midland Naturalist* **85**, 289–328 (1971).

105. Bhardwaj, S. K. & Kumar, P. Photosensitivity in body mass and testicular activity of brahminy myna, Sturnus pagodarum. *Reprod. Nutr. Dev.* **44**, 365–369 (2004).

106. Erickson, M. M. *Territory, annual cycle, and numbers in a population of wren-tits (Chamaea fasciata)*. vol. 42 (University of California Press, 1938).

107. Millikan, G. C. Observations on Galápagos tool-using finches in captivity. *Living Bird* **6**, 23–41 (1967).

108. Bowman, R. I. Morphological differentiation and adaptation in the Galapagos finches. *Univ Calif Publ Zool* **58**, 1–302 (1961).

109. Brown, K. S. & Neto, J. V. Predation on Aposematic Ithomiine Butterflies by Tanagers (Pipraeidea melanonota). *Biotropica* **8**, 136–141 (1976).

110. Areta, J. I. *et al.* Natural History, Distribution, and Conservation of Two Nomadic Sporophila Seedeaters Specializing on Bamboo in The Atlantic Forest: Historia Natural, Distribución y Conservación de Dos Especies Nómades de Sporophila que se Alimentan de Semillas de Bambú en la Selva Atlántica. *The Condor* **115**, 237–252 (2013).

111. Baptista, L. F. Handedness, Holding and Its Possible Taxonomic Significance in Grassquits, Tiaris Spp. *Ibis* **118**, 218–222 (1976).

112. Reed, Chester A. (Chester Albert). *American ornithology, for home and school*. vol. v.5 (1905) ([Worcester, Mass, Charles K. Reed], 1901-[1906], 1905).

113. Bent, A. C. *Life Histories of North American [birds].: Flycatchers, larks, swallows and their allies*. vol. 107 (US Government Printing Office, 1942).

114. Brewster, William. *New England birds : systematic notes*. vol. v.2 (1865).

115. Gross, A. O. Nesting of the Streaked Flycatcher in Panama. *The Wilson Bulletin* **62**, 183–193 (1950).

116. Hudson, W. H. (William Henry). *Birds of La Plata*. vol. 1 (J.M. Dent & Sons ltd, 1920).

117. Safford, R. & Hawkins, F. *The birds of Africa: Volume VIII: The Malagasy region: Madagascar, Seychelles, Comoros, Mascarenes*. (Bloomsbury Publishing, 2020).

118. Nolan, V. Breeding Behavior of the Bell Vireo in Southern Indiana. *The Condor* **62**, 225–244 (1960).

119. Bent, A. C. *Life histories of North American wagtails, shrikes, vireos, and their allies*. vol. 197 (Courier Corporation, 1965).

120. Gill, F. B. Ecology and Evolution of the Sympatric Mascarene White-Eyes, Zosterops borbonica and Zosterops olivacea. *The Auk* **88**, 35–60 (1971).

121. Pistorius, P. A. Grey Heron (Ardea cinerea) Predation on the Aldabra White-throated Rail (Dryolimnas cuvieri aldabranus). *wils* **120**, 631–632 (2008).

122. Moseley, E. L. Blue Heron Colonies in Northern Ohio. *The Wilson Bulletin* **48**, 3–11 (1936).

123. Higuchi, H. Bait-fishing by the Green-backed Heron Ardeola striata in Japan. *Ibis* **128**, 285–290 (1986).

124. Beckmann, C. An Intraspecific Killing in Adult Pacific Reef Egrets (Egretta sacra). *wils* **120**, 422–424 (2008).

125. Morris, A. K. White Ibis eats snake. *EMU* **73**, 73–73 (1973).

126. Martin, J. W. & Kroll, J. C. Hoarding of corn by Golden-fronted Woodpeckers. *The Wilson Bulletin* **87**, 553–553 (1975).

127. Auersperg, A. M. I. *et al.* Social transmission of tool use and tool manufacture in Goffin cockatoos (Cacatua goffini). *Proceedings of the Royal Society B: Biological Sciences* **281**, 20140972–20140972 (2014).

128. Wallace, A. R. *The Malay Archipelago: The land of the orang-utan, and the bird of paradise. A narrative of travel, with studies of man and nature.* (Macmillan and Co., 1869).

129. Borsari, A. & Ottoni, E. B. Preliminary observations of tool use in captive hyacinth macaws (Anodorhynchus hyacinthinus). *Anim Cogn* **8**, 48–52 (2005).

130. Dowsett-Lemaire, F. On the importance of the forest tree Parinari excelsa in the diet of Brown-necked Parrots Poicephalus robustus in Malawi-Zambia. *Bulletin of the African Bird Club* **11**, 139–141 (2004).

131. Best, H. A. The Foods of Kakapo on Stewart Island as Determined from Their Feeding Sign. *New Zealand Journal of Ecology* **7**, 71–83 (1984).

132. Nichols, O. G. From Field and Study: The Port Lincoln Parrot (Barnardius zonarius zonarius) feeding on Lerps at Kalgoorlie. *The Western Australian Naturalist* **14**, 76–77 (1978).

133. MacGillivray, W. *A history of British birds, indigenous and migratory: including their organization, habits, and relations; remarks on classification and nomenclature; an account of the principal organs of birds, and observations relative to practical ornithology ...* vol. v. 3 (London, Printed for Scott, Webster, and Geary, 1837-1852).

134. Csermely, D. & Gaibani, G. Is Foot Squeezing Pressure by Two Raptor Species Sufficient to Subdue Their Prey? *The Condor* **100**, 757–763 (1998).

135. Olver, M. D. Breeding Biology of the Reed Cormorant. *Ostrich* **55**, 133–140 (1984).

136. Cole, C. F. Observations on the Cormorants along the Coast and in the Inland Waters of Victoria. *Emu - Austral Ornithology* **8**, 76–78 (1908).
