# Supplementary material for: Online repositories of photographs and videos provide insights into the evolution of skilled hindlimb movements in birds
Source: Commun Biol. 2023 Aug 15;6:781. doi: 10.1038/s42003-023-05151-z (PMC10427617; doi:10.1038/s42003-023-05151-z)
Supplement: Supplementary file 3 — Description of Additional Supplementary Files [file 42003_2023_5151_MOESM3_ESM.pdf]

Description of Additional Supplementary Files

File name: Supplementary Data 1
Description: Source for each individual media and skill score.

File name: Supplementary Data 2
Description: Behavioral score for each species. This is the source data for all graphs in the paper.
